# A time-responsive tool for informing policy making: rapid realist review

**DOI:** 10.1186/1748-5908-8-103

**Published:** 2013-09-05

**Authors:** Jessie E Saul, Cameron D Willis, Jennifer Bitz, Allan Best

**Affiliations:** 1 InSource Research Group, 982 Thermal Dr., Coquitlam, BC V3J 6S1, Canada; 2 North American Research & Analysis, Inc, 1016 11th Avenue NE, Faribault, MN 55021, USA; 3 Centre for Clinical Epidemiology and Evaluation, Vancouver Coastal Health Research Institute, The University of British Columbia, Research Pavilion, 7th Floor, 828 West 10th Avenue, Vancouver, BC V5Z 1M9, Canada; 4 School of Population Health, The University of Adelaide, Level 7, 178 North Terrace, Adelaide, SA 5005, Australia; 5 InSource Research Group, 4581 Anhalt Rd, Kelowna, BC V1W 1P7, Canada; 6 InSource Research Group, 6975 Marine Drive, West Vancouver, BC V7W 2T4, Canada; 7 School of Population and Public Health, University of British Columbia, 2206 East Mall, Vancouver, BC V6T 1Z3, Canada

**Keywords:** Realist review, Rapid review, Evidence synthesis, Health policy

## Abstract

**Background:**

A realist synthesis attempts to provide policy makers with a transferable theory that suggests a certain program is more or less likely to work in certain respects, for particular subjects, in specific kinds of situations. Yet realist reviews can require considerable and sustained investment over time, which does not always suit the time-sensitive demands of many policy decisions. ‘Rapid Realist Review’ methodology (RRR) has been developed as a tool for applying a realist approach to a knowledge synthesis process in order to produce a product that is useful to policy makers in responding to time-sensitive and/or emerging issues, while preserving the core elements of realist methodology.

**Methods:**

Using examples from completed RRRs, we describe key features of the RRR methodology, the resources required, and the strengths and limitations of the process. All aspects of an RRR are guided by both a local reference group, and a group of content experts. Involvement of knowledge users and external experts ensures both the usability of the review products, as well as their links to current practice.

**Results:**

RRRs have proven useful in providing evidence for and making explicit what is known on a given topic, as well as articulating where knowledge gaps may exist. From the RRRs completed to date, findings broadly adhere to four (often overlapping) classifications: guiding rules for policy-making; knowledge quantification (*i.e.*, the amount of literature available that identifies context, mechanisms, and outcomes for a given topic); understanding tensions/paradoxes in the evidence base; and, reinforcing or refuting beliefs and decisions taken.

**Conclusions:**

‘Traditional’ realist reviews and RRRs have some key differences, which allow policy makers to apply each type of methodology strategically to maximize its utility within a particular local constellation of history, goals, resources, politics and environment. In particular, the RRR methodology is explicitly designed to engage knowledge users and review stakeholders to define the research questions, and to streamline the review process. In addition, results are presented with a focus on context-specific explanations for what works within a particular set of parameters rather than producing explanations that are potentially transferrable across contexts and populations. For policy makers faced with making difficult decisions in short time frames for which there is sufficient (if limited) published/research and practice-based evidence available, RRR provides a practical, outcomes-focused knowledge synthesis method.

## Background

Efforts to improve the connections between research evidence and policy decisions have resulted in a number of sophisticated synthesis tools [1]. Pawson has broadly classified evidence synthesis approaches as being meta-analytical (re-analysing data from numerous studies to arrive at broadly generalizable findings) or narrative (using text-based data extractions to compare study findings as a way for understanding why programs or interventions have certain effects), both of which are commonly employed for informing policy decisions [2]. While Pawson notes the relative advantages of meta-analytical and narrative approaches, each technique also has key limitations: meta-analyses fail to provide understanding of how programs work, while narrative reviews give little insight into how a program may operate in different settings or under different circumstances [3,4]. Limited generalizability of study results for a given context has been addressed as a common concern in evidence-informed public health policy [5,6]. Alternatively, we have realist reviews, which are an approach to synthesising evidence first promoted by Pawson [4].

Pawson notes that the underlying principles of realist approaches are the links between interventions (I), contexts (C), mechanisms (M), and outcomes (O) [4]. In contrast to specific activities included as part of an intervention or program, mechanisms (M) are thought to be the responses by people that are triggered by changes in context (C) [7]. Traditional realist syntheses use these C-M combinations to generate transferable ‘program theories’ that suggest that certain interventions are more or less likely to work in certain ways, for certain people, in certain situations [3,4]. In other words, how does a change in context generate a particular mechanism that in turn produces specific outcomes?

In developing these program theories, realist reviews assume a broad inclusion of evidence, both quantitative and qualitative, which is used to develop an understanding of ‘what works, for whom, in what contexts, to what extent, and most importantly how and why [2,3]?’ Therefore, instead of offering prescriptive advice on what the ‘best buys’ might be for policy makers, a realist synthesis attempts to provide a transferable theory that suggests that a certain program is more or less likely to work in these respects, for these subjects, in these kinds of situations [2,3].

However, realist review methods are not without their difficulties. The iterative, flexible nature of realist reviews does not align well with protocol-driven, standardized processes common to established systematic review methods [8]. As results from a realist review are context dependent, the generalizability of findings to other settings will depend on the operation of similar mechanisms to generate outcomes of interest. Moreover, in completing a realist review, a high level of training and experience is required, which may not be found routinely in government or policy development agencies, academic institutions, or community-based organizations. Perhaps most relevant to our current discussion, realist reviews as outlined by Pawson *et al.*[3,8] can require considerable and sustained investment over time, which does not always suit the time-sensitive demands of many policy decisions. In addition, due to their expansive and exploratory nature, realist reviews can often suffer from ‘scope creep’.

‘Rapid reviews’ have emerged in response to the incompatibility between information needs of policy makers and the time requirements to complete systematic reviews. Rapid reviews provide a way to generate similar types of knowledge synthesis as more comprehensive systematic reviews do, but in a much shorter time period. While some have questioned the validity of rapid reviews [9,10], there remains a need to achieve a balance between comprehensiveness and timeliness for many policy-relevant decisions [11]. Therefore, understanding how this realist approach may be applied in cases where there is limited time and resources to generate a realist-based product that can incorporate research, theory, and practice knowledge and thus meet the demands of real-time policy developers/evaluators, is a valuable contribution to both evidence-informed policy-making and the realist review literature.

‘Rapid Realist Review’ methodology (RRR) has been developed by members of our group as a tool for applying a realist approach to a knowledge synthesis process and producing a product that is useful to policy makers in responding to time-sensitive and/or emerging issues where there is limited time and resources. Where relevant, we compare the RRR methodology to realist reviews generally, or ‘traditional’ realist reviews. While there is no set definition for what constitutes a realist review other than its use of realist logic and constructs, we present ‘traditional’ realist reviews as those that typically engage in a much longer exploration of the literature and ‘testing’ of theories, as well as those that present results within a framework of theory development. While others have begun to describe methodologies for short-term evidence synthesis projects [12], the RRR methodology described here is intended to incorporate the theory specification of a realist review [8] and the boundary clarification aim of a scoping review [13]. (See [11] for an example of an earlier review). This is also consistent with the recently published RAMESES (Realist And MEta-narrative Evidence Syntheses: Evolving Standards) publication standards for realist syntheses, which note that realist reviews need to be focused based on the time and resources provided as well as the questions that need to be answered [14].

In our experience, policy makers have been more interested in knowledge syntheses that highlight possible interventions (I) that could be implemented within a specific context (C) that in turn interact with various mechanisms (M) and produce outcomes (O) of interest. As a result, the emphasis of our reviews has changed over time from one focused on producing transferable theories, to one focused on identifying groups of interventions related to outcomes of interest for policy makers. Our modified review methodology ‘works backwards’ from the desired outcome (*e.g.*, large scale transformation of health systems, sustainable cultural change, etc.), to ‘families of interventions’ (I) that can be implemented to produce those outcomes, supported by a theoretical understanding of the contexts (C) within, and mechanisms (M) by which such interventions operate. In doing so, the RRR methodology focuses less on the development of theory that is transferable across domains, than on the distillation of theory-driven, contextually relevant interventions that are likely to be associated with specific outcomes within a particular set of parameters.

Here, we describe our experience in developing RRR, the key ingredients in making it work, the policy questions to which it is most suited, the strengths and limitations of the method, and the future directions of how this approach may be optimized in practice. In doing so, we provide key examples of RRR projects completed by our group over the past six years.

## Methods

We provide here brief summaries of key examples of reviews completed to date by this team. Table 1 includes the commissioning agencies, title, duration and purpose of each review. For this paper, we also considered key findings and recommendations resulting from each, as well as the strengths and challenges that arose while completing the review. We draw from these examples to highlight the conceptual strengths and limitations of rapid realist review methods and the circumstances under which they offer most value.

**Table 1:** Examples of rapid realist reviews undertaken by our group

**Commissioning agency**	**Title**	**Purpose statement**	**Duration**
Public Health Agency of Canada	Interventions To Increase Organisational Capacity To Address Health Literacy (2012)	The purpose of this project was to conduct a realist review of available literature (published and grey) to summarize what we know and what we don’t know about the development of capacity within organizations to plan, implement, and sustain health literacy interventions. This review did not assess the relative efficacy of health literacy interventions themselves, but rather, attempted to identify what mechanisms and contextual factors improve the capacity of healthcare organizations to address health literacy in the individuals they serve by planning, implementing, evaluating and sustaining interventions.	Six months
Canadian Institutes of Health Research (Partnering organization - Saskatchewan Ministry of Health)	Knowledge And Action For System Transformation (KAST): A Systematic Realist Review and Evidence Synthesis of the Role of Government Policy in Directing Large System Transformation (2010)	The Saskatchewan Ministry of Health has a mandate to support a significant transformation of the provincial health system. Large system transformation refers to systematic initiatives to create coordinated change across organizations working toward shared priorities within specified boundaries. The Ministry recognizes that success hinges on a cultural change based on collaboration, a comprehensive innovation strategy for system redesign, and systems integration. Therefore, they seek guidance -- on such considerations as successful models and strategies, partnership principles (including with patients), monitoring and evaluation -- from a systematic review of knowledge on large system transformation. With all of these considerations, there is an emphasis on the role of government.	Six months
HealthLink BC and QUILTS	Tele-health Contributions to Emergency Department and Discharge Operations (2009)	A rapid review of tele-health contributions to Emergency Department (ED) and discharge operations was conducted to:	Five months
		1. Describe the patient population in selected local ED facilities; identify major drivers of ED utilization, throughput and follow-up; and conduct a rapid review of the effectiveness of tele-health strategies for improving ED/discharge system performance.	
		2. Scan best practices in other jurisdictions and identify potential opportunities for HealthLink BC to add value for regional health authority ED/discharge systems.	
		3. Produce a model of system operations and a framework for ways in which HealthLink BC tele-health services might help to improve performance for ED services, and for discharge planning and follow-up.	
Public Health Agency of Canada	Evidence-informed public health policy and practice through a complexity lens (2007)	This RRR was commissioned to summarize what is known about systems to support evidence-informed policy and what is not known. The review focused on frameworks most applicable to public health, with an emphasis on chronic disease prevention and healthy living. The review process also initiated an international collaboration with a focus on system development for evidence-informed public health.	Two months
HealthLine Services BC	Tele-health in Support of Chronic Disease Management (2007)	The purpose of this project was to provide HealthLine Services BC (HLSBC) with a review of evidence and best practices for health line support for chronic disease management.	Six months
Vancouver Coastal Health	Population-based Framework for Chronic Disease Self-management Support (2006)	The overall goal of the project was to identify the most current view on effective models for self-management and self-management support, and to provide decision makers in the policy and practice community at Vancouver Coastal Health with a practical understanding of evidence-based interventions, within a population-based framework for self-management support.	Three months
NIH Office of Behavioral and Social Science Research and the Canadian Health Services Research Foundation	Interorganizational partnerships (2006)	To provide a RRR of evidence-based principles for effective interorganizational collaboration and partnership, as the principles relate to two contexts: the collaborations in which health research funders engage; and the forms and processes of linkage within universities and their community partners to promote interdisciplinary health research and effective knowledge exchange. The review also summarized the existing evidence to identify aspects of interorganizational partnerships (a) where sufficient evidence exists to conduct a full systematic review, and (b) where insufficient evidence exists to articulate the further primary research that is necessary.	Two months

### Methods of realist review

As Pawson has described elsewhere [3], key stages and tasks for a realist review include the following steps: identifying the review question, searching for primary studies, quality appraisal, extracting the data, synthesizing the data, and disseminating the findings (see [3], chapter 4). However, while Pawson has stated that realist reviews need to be focused, the methodology he has described has not been explicit about how to focus the review, particularly for cases where a policy recommendation is required quickly. In response to the need for a time- and resource-sensitive review process, we have modified and expanded on Pawson’s original methodology, including the explicit engagement of content experts drawn from stakeholders. While engagement of experts and stakeholders is a common strategy for traditional realist reviews, we have incorporated it within the RRR method explicitly as a means to streamline the review process. Other variations that are distinct from more traditional realist reviews are noted below.

1. Development of the project scope: clarifying with the knowledge users the content area of interest for the review. As with any type of realist review, this step is critical in ensuring a feasible review process, regardless of the desired timeline.

2. Development of specific research questions: once the project scope has been narrowed, discussing the specific questions that knowledge users are most interested in answering. Refining these questions to ensure that there is enough evidence to be able to answer them, at least in part. As with step 1 above, this is a critical component of any type of realist review.

3. Identification of how the findings and recommendations will be used: this includes formulating a purpose statement that helps identify how the findings of the review will be used by the target audience. Utilization of review products is a key element in the RRR methodology, and one which systematic reviews often do not address. While traditional realist reviews may incorporate this step as well, it is not explicitly included in the RAMESES publication standards for realist reviews [14]. This could be considered part of step 2 above, although it has allowed us to focus our reviews more clearly with the knowledge users in mind throughout the searching, extraction, and synthesis process.

4. Development of search terms: collaboratively identify terms likely to be relevant to the project scope, purpose, and research questions.

5. Identification of articles and documents for inclusion in the review (both published and grey): begin with a list of documents as identified by knowledge users and content experts. In addition, use the search terms to iteratively generate lists of documents that may be included in the review.

6. Quality review: narrow the search terms based on the results that are most relevant to the review topic. Simultaneously, poll the knowledge users and external content experts to identify documents (published and grey) of key importance for the review. We explicitly acknowledge that a search using the RRR methodology will not be comprehensive. Polling knowledge users and content experts to identify key articles accelerates our search process. This, combined with the validation step (#8 below), helps ensure that we are not missing significant sections of the literature with our abbreviated search.

7. Extraction of data from the literature: using an extraction template, pull out elements from documents that can help answer the research questions. Data are extracted using identical methods to a traditional realist review. Findings are analyzed to build a form of realist program theory that addresses the agreed focus and scope of the review. As stated earlier, policy makers tend to be more interested in families of interventions that can change context (C) in ways that interact with mechanisms (M) to produce outcomes (O) of interest, and less interested in program theories. However, development of these program theories is essential for understanding the C-M interactions that can then help explain why these families of interventions will or won’t work in the policy maker’s setting.

8. Validation of findings with content experts: once program theories have been generated, they are reviewed by content experts who have direct experience in the field to ensure that they represent the learnings of practitioners, and to fill any gaps that may have been left by the published literature.

9. Synthesis of the findings in a final report. The report is formatted in a way intended to meet the needs of the knowledge users, based on the results of step 3 above, and the findings produced by steps 7 and 8.

10. Dissemination of results: working with the knowledge users to apply the findings through policy recommendations, further knowledge gathering and synthesis, or evaluation of knowledge application. Because findings are typically presented to policy makers with a focus on families of interventions that can be implemented within their context, they can be easily acted on. Program theories are presented as a way to understand how changes in context may interact with mechanisms to produce outcomes of interest, rather than as the primary findings. Program theories also help describe potential unintended consequences resulting from changes in context and their resultant interactions with mechanisms.

These ten steps are similar to the procedure for a Cochrane or other systematic review, although they fundamentally differ due to their iterative nature (*i.e.*, steps 1 through 7 may be revisited iteratively throughout the RRR process) and the rapidity with which they are conducted. The primary differences involve the realist philosophical approach to how the extraction, analysis and synthesis are completed (focusing on how context interacts with mechanisms to produce outcomes), and the involvement of knowledge users in shaping the final product.

It is increasingly a necessity to engage knowledge users in order to receive funding to conduct reviews of all types. This fits well with the RRR design, and also constitutes one of its critical strengths. In particular, the involvement of a local reference group and expert panel facilitate both more efficient identification of key materials to include in the review, and sufficiently robust findings. Involvement of knowledge users and external experts also increases both the usability of the review products, as well as their link to current practice. Our engagement of a local reference group and an expert panel is consistent with the recommendations put forth by Lavis *et al.* to create a ‘dialogue that allows the data and research evidence … to inform and be considered alongside the views, experiences, and tacit knowledge of those who will be involved in, or affected by, future decisions about the health system problem’ [15].

At all stages of the process, a local reference group is engaged to ensure that the project will produce results that will be relevant for the context in which they will be used. The reference group typically includes representatives of the funding organization, as well as knowledge users (the target audience for the findings of the review). As Keown *et al.* have shown, the potential benefits of including stakeholders in the process of a review include increased relevance, clarity and awareness of review findings [16]. Van de Ven [17,18] provides a similar view for coproduction that he calls ‘engaged scholarship’. Given the explicit intention of the RRR methodology to provide results that can be used by policy makers, these benefits become critical to the success of a project.

In addition to the local reference group, the RRR methodology identifies an expert panel made up of researchers and practitioners, actively engaged in conducting work in the content area for the review, who are in the process of negotiating the interplay between research, practice and policy. Engagement of an expert panel provides several benefits, including focusing the review scope; streamlining the search process; filling gaps in the initial problem definition, search process or synthesis of findings; and ensuring appropriate interpretation of results.

While these experts are often scattered internationally, several factors have allowed us to rapidly convene such panels within a matter of weeks from project initiation. First, knowledge users often have connections to one or more content experts for the topic, given their interest and involvement with the topic. During initial discussions of project scope and research questions, collectively we identify experts who already have connections to the project team or knowledge users. Initial invitations are extended to this small group who are fit for purpose for a given review. Additional targeted invitations are extended to those publishing within the domain of interest. Interest and engagement can often be enhanced by further invitations coming from, or mentioning, experts who have already agreed to participate. Finally, the time commitment required by expert panellists is deliberately minimized, and highlighted in the invitation. If starting from scratch, identification and engagement of a group of content experts can be enormously time consuming. However, we have been able to circumvent the process of identification, introduction, invitation and engagement due to our ability to draw on first- and second-degree network connections.

The initial search places a special emphasis on grey literature that may not be easily accessible through traditional search methods, especially for cases in which the published literature on the selected topic may be of limited relevance to the specific research question at hand. Inclusion and exclusion criteria are developed to assist with selection of documents to review further. Based on these criteria, a screening tool is developed that is used to screen abstracts that are identified using the initial search terms. As the search progresses, search terms and inclusion/exclusion criteria are modified iteratively with the goal of identifying the documents most relevant to the review topic without missing many that might be important to include. The expert panel is asked to contribute a list of the most relevant articles at the start of the search to streamline the process. The importance of the expert panel’s and reference group’s help in the search process cannot be underestimated with respect to conducting a rapid review.

Once an initial list of documents has been identified, pilot extractions are conducted with all members of the review and extraction team. The extraction template includes fields such as ‘theoretical foundation or conceptual framework’, ‘facilitators/barriers to success of the project’ (*e.g.*, contextual changes that helped or hindered the triggering of mechanisms to produce both intended and unintended outcomes), and ‘interactions between context and mechanisms’. Template fields are modified and tailored to meet the needs of each individual review project. A series of calibration exercises consist of comparing the extractions of the various team members for level of detail, identification of relevant themes, and individual style for presentation of findings. Between three and five articles are typically reviewed in this fashion. Further extractions are conducted by individual team members. Additional consultations are conducted as needed throughout the review process. As documents are extracted, forward and backward citation searches are conducted on key articles. Additional documents are added to the review list throughout the process. Because the timeline for each rapid review is typically tight (six months or less), there is a limited window of opportunity to add documents to the review. This emphasizes the importance of accelerating the review process with several key documents that can be used for forward and backward searching.

After the initial round of extractions is conducted, the review team summarizes key themes and findings, focusing on contextual factors and mechanisms that impact outcomes, and how context and mechanisms interact. Typically, a matrix is created listing contextual factors and mechanisms. Findings are grouped by similar contextual changes and how they trigger mechanisms to produce outcomes (demi-regularities). These groupings are regularly shared and discussed within the review team to ensure validity and consistency in the inferences made, although due to the time restrictions, this typically occurs twice or three times during the review. Findings are reviewed simultaneously with the local reference group. Based on the preliminary findings, the project questions, inclusion and exclusion criteria, and the fields for the extraction template are adjusted as needed. Following this process, the extraction process is completed, and a draft of key findings is presented to both the local reference group and expert panel. Feedback is obtained to ensure relevance to the local context of knowledge users, consistency with the body of published and grey literature, and consistency with the current practice and tacit knowledge of the content experts. Feedback is used to expand on the findings and tailor the synthesis for the final report.

Team members needed to complete a Rapid Realist Review include:

1. Project manager, responsible for preparing internal project documents, coordinating the dialogue and managing a pre-determined set of requests for the reference group and expert panel (providing feedback at each stage of the process from question development to review of the final report), consolidating feedback, maintaining the timeline, budget and other duties;

2. Local Reference group (including client representatives) and expert panel (ideally four to six individuals for each group);

3. Librarian (or information specialist) to lead on document searches;

4. Review team (two to four individuals who screen abstracts, read selected documents, and perform extractions);

5. Synthesis lead to oversee the review process and play a main role in synthesizing information;

6. Academic or research lead.

The composition of the review team is critical for the RRR methodology. All team members must be well-versed in the realist philosophy that serves as the theoretical underpinning for realist reviews. In addition, the academic or research lead and synthesis lead must not only be familiar with realism and realist approaches to synthesis reviews, but must also have participated in a realist review process previously. It is critical that the review team members approach the project not from a positivist, linear, cause-and-effect perspective, but rather from the perspective that mechanisms for change will always be context-dependent. It is this grounding in the realist philosophical tradition that distinguishes realist reviews from other types of systematic reviews. At the same time, it is essential that the review team have a clear sense of the scope and purpose of the review to ensure maximum utility and application of the results for each project’s unique audience. Familiarity with the context in which the results will be used (*e.g.*, policy development) is also helpful.

Team composition differs for an RRR as compared to a traditional realist review in that the project manager must be disciplined and able to keep a large complex process moving along according to schedule. The local reference group and expert panel must be prepared to provide feedback in a timely fashion, as this can often be a limiting factor in how quickly a review can be completed. And finally, the synthesis lead must be able to review large quantities of information efficiently, distil it into easily digestible components for review by the reference group and expert panel, and quickly incorporate feedback while keeping the intended uses of the final product in mind. The reference group (knowledge users) must also be clear about their priorities, which can help the review team make decisions about the review process, particularly as the project deadline approaches. Panel involvement varies in intensity over time. Clarity of purpose and timelines, and a willingness of panellists to respond within the allocated time frame, are essential components of a project as well.

## Results

Table 1 provides details of the RRRs completed by this team to date. Agencies commissioning RRRs have included Federal Government Agencies (*e.g.*, Public Health Agency of Canada, U.S. National Institutes of Health), Provincial Health Ministries (*e.g.* Saskatchewan Ministry of Health) and associated initiatives (*e.g.*, Healthlink BC), regional health authorities (*e.g.*, Vancouver Coastal Health Authority), and key research funding bodies (*e.g.*, Canadian Institutes of Health Research). In a number of instances, reviews have been funded by research granting agencies for work to be completed in partnership with identified collaborators.

There is considerable variation in the topical focus of each RRR included in Table 1. Reviews often have been commissioned to synthesize information on complex, system oriented issues, such as large system transformation, improving organizational competencies for addressing health literacy needs, enhancing interorganizational partnerships, and improving evidence-informed health policy as well as bettering the people-centredness of health authorities. In addition, a number of reviews have focused on specific clinical specialties, domains or services, such as tele-health interventions, emergency department discharge practices, and optimal care models for chronic disease management programs.

As outlined in Table 1, RRRs may be completed for a variety of purposes. Two broad purpose domains appear to emerge, with RRRs used either to inform the development of practical solutions to problems faced in care delivery, or to develop a more conceptual understanding of how to organize ideas or frameworks for refining high level strategic thinking (Table 1). For the former, RRRs have been used to synthesize best practices for specific service provisions, such as evidence-based tele-health initiatives or an inventory of self-management interventions for chronic disease patients. More conceptual applications have produced ‘simple rules’ for guiding large system transformation, syntheses of frameworks for supporting evidence-informed policy-making and strategies for fostering organizational partnerships. Methodologically, RRRs have offered decision makers a tool for merging sources of evidence including published literature, grey literature and expert opinion, and as such, have provided knowledge users with the ability to make sense of how and why they might expect certain outcomes to occur, and the role of context in these outcomes. Further, they have helped knowledge users determine if sufficient substrate exists for other structured review exercises. In doing so, RRRs have proven useful in providing evidence for and making explicit what is known on a given topic, as well as articulating where knowledge gaps may exist.

From the RRRs completed to date, findings broadly adhere to four (often overlapping) classifications: guiding rules for policy making (*i.e.*, evolving patterns of decision-making that can be adapted based on new knowledge or contextual factors); knowledge quantification (*i.e.*, the amount of literature available that identifies context, mechanisms and outcomes for a given topic); understanding tensions/paradoxes in the evidence base, as when similar changes in context trigger similar mechanisms and produce divergent outcomes, or when changes in context trigger different mechanisms to produce the same or different outcomes; and, reinforcing or refuting beliefs and decisions taken. See Table 2 for case studies that include detail on specific findings for two reviews. RRR provides a tool for developing rules for guiding decision-making, such as the advantages of investing in both top-down and distributed leadership, as well as on-going performance measurement in large system transformations (*e.g.*, Knowledge And Action For System Transformation (KAST) review), or the need for innovative inter-organizational investments for improving the links between knowledge creation and adoption. While these guiding rules can inform the development of strategic directions, they can also be used as tools to confirm or dispel existing beliefs; for example, confirming the superiority of multi-faceted chronic disease prevention programs compared to non-multi-faceted approaches within a specific context for particular populations, or identifying that large scale changes in Canadian health systems at this moment in time require engaged physician and patient groups. Because they highlight I-C-M interactions, they also make apparent situations when similar types of interventions would not result in their intended outcomes due to contextual differences triggering alternate mechanisms. RRRs make explicit the amount of literature readily available for review, and in the case of a limited evidence base, can draw on the input of the expert panel in understanding the absence of literature and subsequently developing directions to take in sourcing other relevant literature for the review. In addition to understanding when little is known on a given topic, RRRs also highlight where tensions or uncertainties exist in an evidence base, such as what the precise characteristics of an effective network might be for a given purpose within a specific geographic, political and temporal context, or the differential effectiveness of tele-health methods for chronic disease self-management for populations within varying contexts.

**Table 2:** Case studies

**Project title:**	Knowledge And Action For System Transformation (KAST): A Systematic Realist Review and Evidence Synthesis of the Role of Government Policy in Directing Large System Transformation	Interorganizational Partnerships
**Partner:**	Saskatchewan Ministry of Health	National Institutes of Health - Office of Behavioral and Social Science Research and the Canadian Health Services Research Foundation
**Funder:**	Canadian Institutes of Health Research	Same as partner
**Length of time:**	Six months	Two months
**Published article:**	Best A, Greenhalgh T, Lewis S, Saul J, Carroll S, Bitz J 2012. Large system transformation in health care: A realist systematic review and evaluation of its usefulness in a policy context. The Milbank Quarterly, 90(3): 421–456.	Riley BL, Best A. (in press). Stakeholders, organizational partnerships, and coalitions. In S. Kahan, A. Gielen, P. Fagan, & L.W. Green (eds). Health Behavior Change in Populations: The State of the Evidence and Roles for Key Stakeholders. Baltimore, MD: Johns Hopkins University Press.
**Purpose:**	The Saskatchewan Ministry of Health has a mandate to support a significant transformation of the provincial health system. Large system transformation refers to systematic initiatives to create coordinated change across organizations working toward shared priorities within specified boundaries. The Ministry recognizes that success hinges on a cultural change based on collaboration, a comprehensive innovation strategy for system redesign, and systems integration. Therefore, they seek guidance -- on such considerations as successful models and strategies, partnership principles (including with patients), monitoring and evaluation -- from a systematic review of knowledge on large system transformation. With all of these considerations, there is an emphasis on the role of government.	To provide a RRR of evidence-based principles for effective interorganizational collaboration and partnership, as the principles relate to two contexts: the collaborations in which health research funders engage; and the forms and processes of linkage within universities and their community partners to promote interdisciplinary health research and effective knowledge exchange. The review also summarized the existing evidence to identify aspects of interorganizational partnerships (a) where sufficient evidence exists to conduct a full systematic review, and (b) where insufficient evidence exists to articulate the further primary research that is necessary.
**Questions asked in the review:**	1. What are the key mechanisms or social processes that influence or drive successful large systems transformation in the health care sector?	1. What are the evidence, experiential and theory-based critical success factors for increased interorganizational collaboration?
	2. What are the contextual factors that have the most impact (positive or negative) on large systems transformation efforts in the healthcare sector?	2. What is the nature of the evidence, and what is known about external validity and generalizability?
	3. Are there identifiable ‘transition’ points in large systems transformation efforts? If so, how do the key mechanisms and contexts interact to produce these changes?	3. What are the best indicators to monitor collaboration and outcomes a) within the research funder context, b) within the university/institutional (researcher performer) context, and c) within the context of the users of research knowledge (research user)?
	4. What is the role of government or government-like entities (these need definition or at least examples) in large system transformation efforts?	4. What are the strategies most likely to generate buy-in of key influentials in each context for strategic plans and new initiatives?
		5. What does the literature say about the challenges or constraints most likely to affect success in each context?
		6. What does the literature say about stages of the development of collaborations and the most effective tactics at each stage?
**Resulting evidence statements**	(In the full report, each has an associated list of contextual factors and related mechanisms):	From a synthesis of literature on the subject, the review identified a number of key factors critical to realizing the full promise of ‘the collaborative advantage.’
	1. Large system transformation in healthcare systems requires both top-down leadership that is passionately committed to change, as well as distributed leadership and engagement of personnel at all levels of the system.	1. **Clear common aims**. It often takes time and cycling through the various stages of direction setting, action, and trust building, to develop the overarching partnership-level goal, common language, and aims necessary to enable and sustain a productive partnership.
	2. Measurement and reporting on progress toward short- and long-term goals is critical for achieving effective and sustainable large system transformation.	2. **Trust**. An essential foundation for any partnership, trust builds on itself over time, often starting with modest, low-risk initiatives.
	3. Consideration and acknowledgement of historical context will help avoid unnecessary pitfalls, and increase buy-in and support from system stakeholders.	3. **Leadership**. Effective interorganizational collaboration requires sustained and engaged leadership, and a shift in leadership/management style from ‘command and control’ to facilitating and empowering, and from delegation to participation.
	4. Large system transformation in healthcare systems relies on significant physician engagement in the change process.	4. **Sensitivity to power issues**. Each partner in a collaborative brings different resources and strengths. Effective collaboration requires acknowledging these differences and taking care in negotiating expectations and the ground rules for decision-making.
	5. Large system transformation that aims to increase patient-centeredness requires significant engagement of patients and families in the change process.	5. **Membership structures**. Shared understandings about what the collaboration involves, and formalized rules, roles and structures enable participation. Both governance and task structures are important. The evidence shows the need for effective coordination infrastructure with agreed-action strategies, and sufficient resources, capacity, and role clarity to support good communication and management functions. Because membership is often dynamic and changing, continuing work is essential to sustain the shared understanding and common focus. Effective coordination structures also help speed the uptake of innovations.
		6. **Action learning**. Effective collaborations continuously improve through feedback loops and reflective shared learning.
**Strengths of the review:**	Much of the success of this review was due to the timely topic, which inspired commitment from the reference group and expert panellists. Key to this was the direct involvement of key decision makers in the Saskatchewan government, which ensured relevancy of the final product and maximized the potential for uptake of the findings. The project was designed for and succeeded in blending practice knowledge with research knowledge. Within the design, this review was unique in the use of a consensus network to validate the findings. This network was made up of national and international experts in the area; using an online survey, they were asked to comment on the five evidence statements and reflect on how well these statements compared with their understanding and experience. Out of 100 people invited, 50 % took part. Considering the voluntary nature and the length of the survey (it took people on average 30 minutes), we deemed this a very good response rate. The consensus network was particularly useful given the literature base was relatively sparse. Also contributing to the success of the project was the opportunity to link the final workshop with another major CIHR initiative, providing an additional opportunity for shared learning and partnering.	This review was positioned to inform a strategic planning initiative for OBSSR; as such it was an important support to future work for them and was applied in practice almost immediately. This review began with a key set of articles that were provided by the research team and expert panellists, thus expediting the process. Articles were added as we came across them through citation searching or from the panellists over the duration of the project. As is the case in all successful RRRs, the expert panel was engaged and committed to the topic.
**Challenges for the review:**	As is often a reason for choosing a realist review methodology, the amount of published and grey literature on the topic was scarce. Remedies to this challenge were in-depth and on-going consultation with a reference group and an expert panel, further enhanced by the use of the consensus network. An additional challenge was the fact that the findings were intuitive, and did not appear to provide ‘novel’ solutions or courses of action to the decision makers in the Saskatchewan Ministry of Health. However, they were pleased to have formal confirmation of what they already knew. Considering tacit knowledge often runs ahead of research knowledge, this can be a typical of many reviews, rapid and otherwise.	The review had to be done within a two-month timeframe with limited resources. This meant no time or money to include a key word literature search. However, due to the research team’s prior work and the expertise from the panel, the project was initiated with a solid set of articles for review, and completed within the timeframe allocated for the project.

The RRRs cited in this analysis have a number of strengths. Engaged members of both the expert panel and reference group ensure rigorous methods by providing an extended body of expertise and experience, and validating findings and program theories in an iterative way. In addition, the participation of the reference group increases the local utility of review findings. Moreover, the results speak to the value of diverse data collection methods, building on the literature through methods such as surveys and focus groups. In addition, reference groups tend to include (or provide links to) key decision makers in commissioning agencies, leading to closer connections between knowledge producers and users. As such, the RRRs completed to date have generally performed well as tools for responding to time-sensitive policy issues.

As with any review process, there are challenges to the RRR process. Ensuring that the scope of the review remains contained and the questions definable can be especially important and difficult in the RRR process. Like all reviews, difficulties may emerge when limited published or grey literature exists, where the quality of included studies is poor, or where the findings of studies are heavily contextualised to particular settings or times. Forming the membership of the reference group and ensuring sustained involvement are challenges for all reviews, particularly in rapidly changing political environments where membership of the reference group may change during the course of a given project.

## Discussion

The RRR method outlined here and a more traditional realist review share numerous similarities (see Table 3). Both are designed to be responsive to local policy needs. Both are grounded in realist logic and constructs, explicitly attempting to understand the interactions between contexts, mechanisms and outcomes. However, they also have some key differences, which allow policy makers to apply each type of methodology strategically to maximize its utility within a particular local constellation of history, goals, resources, politics and environment. In particular, the RRR methodology is explicitly designed to engage knowledge users and review stakeholders to narrowly define the research questions, and to streamline the review process, allowing the entire review process to be completed within three to six months (see [11] for an example). There are limitations associated with reducing the time available to complete such a review, as are discussed below. However, some policy decisions are required in a very short time frame (six months or less), thus making a methodology that can gather and synthesize the maximum amount of evidence in a compressed time period extremely useful.

**Table 3:** Comparison of RRR and ‘traditional’ realist review methods

**RRR method**	**Full realist review method**
▪ Examine interactions between mechanisms, context and outcomes	▪ Examine interactions between mechanisms, context and outcomes
▪ Short-term (3- to 6-month turnaround)	▪ Longer term (12- to 24-month turnaround)
▪ Responsive to local policy needs	▪ Reponsive to local policy needs
▪ Results are utilization-focused	▪ Results are utilization-focused
▪ Includes both reference group and expert panel	▪ Likely includes a reference group (or something similar); there is no standard for involvement of an expert panel
▪ Involvement of expert panel allows for conclusions and recommendations to be developed from an emerging/nascent literature	▪ Includes both published and grey literature, search process is comprehensive (until the point of theory saturation), and program theories can be developed over a period of months.

The cost implications for the rapidity of the review process are difficult to quantify, since the bulk of our work is with RRR, thus prohibiting a good comparison within our own team for rapid vs. traditional realist reviews. We surmise that the skillset required for the review and synthesis increases the cost for personnel, although the specific relative costs will vary by project, content area, and individuals involved. While the overall length of time for RRRs will decrease costs overall, project management costs per month are likely to be greater for RRRs than for longer realist reviews, given the necessity to interact frequently with the reference group and expert panel, and the need to obtain timely responses from them.

As discussed, the RRR method outlined here involves both a local reference group and an expert panel. A traditional realist review likely engages a group equivalent to a local reference group, but to our knowledge no expert panel is engaged as a standard part of the realist approach to knowledge synthesis. Among realist reviews more generally, ‘advisory groups’ are quite common, and often serve the role of a merged ‘reference group’ and ‘expert panel’. We have found it useful to separate out the ‘expert panel’ into its own functioning unit. To make the engagement process more efficient, asking the reference group to focus on the utility of the findings, and their relevance to the work at hand within a local context, has worked well. The expert panel is similarly asked to focus its efforts on the validity of the findings and our interpretation of the evidence. The engagement of experts allows for at least two important contributions: firstly, it provides a validation process for findings that have been generated from an expedited review process. In the RRR method, the search strategy is intended to be robust, but not comprehensive. Due to the time constraints, it is fully acknowledged that not all references related to the research question, or even all that will produce theoretical saturation, will be identified and included in the review. However, by engaging the expert panel in the searching stage of the project, we can accelerate the search process, and at the same time be reasonably assured that any key theoretical contributions within critical articles, reports, or other resources (including those in the process of publication) will be identified and included. Secondly, review of preliminary findings by the expert panel validates results of the review, ensuring that the interpretation of the literature is consistent with the experiences of those currently engaged in work around the review topic. The intent of incorporating an expert panel is to integrate the lessons learned from the literature with those of identified content leaders who have experience working to integrate knowledge from research, practice and policy perspectives, and to build program theories based on that combined knowledge.

The RRR method also differs from a more traditional realist review in the role of theory, and specifically the presentation of theory in the final synthesis product. A ‘traditional’ realist review explicitly grounds the review process in theory; it produces a narrative summary of the interactions between context, mechanisms, and outcome configurations [8]. The reviewer then constructs one or more ‘middle range theories’ (*i.e.*, ones that ‘involve abstraction… but [are] close enough to observed data to be incorporated in proportions that permit empirical testing’ [19]) to account for the configurations. The literature is then explored to ‘test’ these theories based on emerging findings from the review. In the RRR method, results are generated through a very similar process; however, theory-building is viewed as a supportive process to identifying families of interventions (I) and explaining why they would produce outcomes of interest (O) by generating specific changes in context (C) that would then trigger particular mechanisms, rather than the primary result. In some cases, however, the content area for the review is nascent enough that there is insufficient published literature to generate fully developed theoretical frameworks in which to ground the review. In those cases, the local reference and expert panels assist in producing and refining program theories that describe the current best thinking about how programs should work in different contexts. In either case, the focus of the review team is to produce generally accepted explanations among policy makers and content experts for what works within a particular set of parameters, rather than aiming to produce explanations that are potentially transferrable across contexts and populations. The distinction may appear to be semantic, but its impact is very real for both process and product of the review.

### Strengths of the rapid realist review process

Reviewing the similarities and differences between the RRR and more traditional realist review methods highlights several strengths of the RRR approach. Similar to realist reviews more generally, the RRR method is time efficient, and may in fact be more amenable to time limitations than other types of realist reviews (due in part to the increased involvement of the expert panel). RRR is grounded in local context, with explicit, extensive, iterative engagement with a local reference group comprising representatives of potential ‘knowledge users’. The reference group provides feedback on the project at critical times, including when developing project scope and research questions, identifying key literature, and interpreting results (‘Do these findings and recommendations make sense for our context? Will we be able to use these results?’). As such, the RRR method is responsive to local policy needs and is therefore adherent to the goals of a realist approach to knowledge synthesis [3]. As a result, while the RRR method is adaptable to scale, it tends to operate most effectively when reviews are commissioned by those who have authority and power to enable change, where the policy questions to be answered map clearly onto the jurisdiction that is responsible for effecting policy change, and where key stakeholders from that jurisdiction who will be putting policy changes into practice are represented on the local reference group (as occurred in the KAST review; see Table 1). When reference groups include (or provide links to) key decision makers in commissioning agencies, this leads to closer connections between knowledge producers and users.

Similar to traditional realist reviews, the RRR methodology also works well in situations where there is a small but emerging body of evidence on which to base policy decisions. The use of the expert panel allows for incorporation of knowledge-in-the-making into policy recommendations, as well as the practice knowledge of those who have been engaged in application of research findings to a policy context. For example, in the review of methods to increase organizational capacity to address health literacy issues within healthcare organizations, the actual published literature specifically relating to the review topic was limited. As a result, the expert panel helped frame the findings significantly to be consistent with what they had experienced in practice. This enhanced the value of the final report for the knowledge users.

Finally, the RRR methodology allows for the ability to scale the project based on the time and resources available. For example, the evidence informed public health policy and practice review consisted primarily of a literature review and synthesis with no reference group. On the other end of the spectrum of review complexity, the large system transformation review (KAST) included a much broader and more extensive consultation with content experts. While this was not critical to the success or usefulness of the final report, it added to the nuanced contextualization provided in the final report. Key to the KAST project, the report commissioners explicitly requested recommendations for government action that could enhance the likelihood of success for large system transformation projects. Having such a request made it easier to elicit useful feedback from the content experts that were surveyed for the project.

### Limitations of the rapid realist review process

While the RRR method has several strengths and advantages, as noted above, there are also several limitations to the RRR approach.

The RRR method explicitly uses an expedited search process. This is an advantage with respect to the faster turnaround time for the finished review, but it may also result in certain resources/references being missed, potentially introducing a source of bias. However, the effects of this potential limitation are buffered by the engagement of the expert panel. Experts validate the findings to ensure that critical elements are not missed, and that nuances from emerging practice are included.

The short time frame in which RRRs are typically conducted can make it difficult to fully theorize the mechanisms that are identified, as well as the interactions between context, mechanisms and outcomes, as is at the core of a full realist review. This may consequently limit the generalizability and potency of findings. Therefore, policy makers interested in sharing results more broadly may need to consider the inclusion of other examples of when the same (or similar) mechanism is in operation, though this might be an intervention that has not been labelled as being the ‘same’ as the one under consideration. However, this is unlikely to be an impediment to the actual utilization of the review findings in practice. Working with the local reference group throughout the project ensures that the project scope, research questions, and format of project outcomes will meet the needs of those who will use the information.

Given the time-intensive nature of the RRR review process, as well as the critical role of the expert panel, it is unlikely that government agencies or departments will have the in-house capacity to complete these reviews. Adding the RRR method to a policy maker’s toolkit will routinely require external input from expert teams. However, this may actually be an advantage; it allows expert methodologists to be engaged at specific times in order to answer specific questions, thus targeting resources strategically. One critical advantage is the experiential learning that results, deepening the systems thinking skills required for effective change. The relative strengths are brought to bear in ways that maximize the allocation of resources, time and talents, making such a process efficient as well as effective.

Forming the membership of the reference group and ensuring sustained involvement are challenges for all reviews, particularly in rapidly changing political environments where membership of the reference group may change during the course of a given project. However, the role of the reference group in the RRR methodology allows key agency or government staff to be engaged in the process without requiring excessive time commitments. Similarly, the engagement of expert panels does rely on panellist availability and willingness to contribute their time and ideas. Providing honoraria for expert panellists, and/or providing opportunities for them to be involved in dissemination of the work through conference presentations, papers, or other avenues, can make it easier to engage the right experts at the right time.

A final potential weakness can result from the requirement that all members of the review team be well versed in realist review methodology, while at the same time commissioners of realist reviews (such as policy makers) do not share the same deep understanding of the methodology and its value for policy. In our experience, policy makers tend to be more concerned about what they are able to accomplish within one specific context (*e.g.*, a province) rather than adding to the knowledge-base of middle-range theories. While they need to understand C-M-O interactions to be able to identify potentially effective interventions, their focus tends to be more in intervention (I) – outcome (O) links. We see a growing interest in the how and why interventions work or don’t work for various audiences, but understanding the program theories that underlie success or failure of interventions tends to be lower on the list of priorities than producing results. As a result, our reports have had to highlight I-O links, and use the program theories we developed to help explain why those interventions might work well in a given context for specific populations.

### Potential risks to the impact of rapid realist reviews

The RRR methodology was explicitly developed to better meet the time-sensitive needs of policy makers for coherent, focused and relevant syntheses of knowledge to develop evidence-informed policies. There are, however, threats to the ability of intended knowledge users to put the findings into practice. While these risks are applicable to any similar review, the underlying purpose of the RRR methodology makes threats to the use of findings particularly relevant. These threats are three fold:

1. Lack of alignment between the policy question and the composition/jurisdiction of the reference group. If those engaged as the local reference group aren’t the ones who will be able to use the results, the process will have significant challenges.

2. Lack of sufficient time to conduct the review, or barriers that prevent the process from proceeding according to schedule. As discussed above, rapid feedback from both reference group and expert panel members is necessary for conducting rapid realist reviews. If competing time commitments prohibit such a level of engagement, the project will lose its ability to produce results in a timely fashion, the quality of the review may be reduced, or the utility of the results may be lessened.

3. Lack of continued engagement and evaluation of the impact of RRRs. Ideally, RRRs would generate evidence-based recommendations for policy action. These actions would be implemented and evaluated to determine their impact. Additional work needs to be done to demonstrate and document the impact and value of the RRR methodology. A lack of engagement or interest in these areas by policy makers will make it more difficult to continue to make the case for the use of such methods in the future.

### Future work

Future work is needed in several areas to support and enhance the value of the RRR methodology in developing evidence-informed policy. First, as noted above, additional measurement is needed to document the outcomes and impact of RRRs. Conducting qualitative research and evaluation with the users of products developed using RRR methods will add enormously not only to the field of implementation science, but will also develop a more robust toolkit for policy makers facing decisions in a context of uncertainty. Second, and relatedly, work is needed to develop robust criteria with which the impact of RRR methods can be assessed. Third, a method is needed to explicitly document the boundaries for findings produced with RRR methods. While results of RRRs typically are presented to stakeholders within one specific context, a method is needed for identifying the limits of the review in terms of time period, geographical area, populations, and environmental factors that might limit the generalizability of results. Finally, a structured program of evaluation needs to be developed that guides this process and that is helpful to users as well as review methodologists. Currently, all assessments of impact are largely based on user feedback. Following completing of reviews, a lag-time is required to enable actual action on review recommendations. The ideal timeframe, process, and methods for assessing impact and outcomes are areas for future research and collaboration.

## Conclusions

Rapid Realist Review offers a useful tool for addressing time-sensitive policy decisions. The RRR methodology provides a way of handling the tight time limits and rapid turnaround necessary that often characterize policy decisions. The risks to the rapid nature of the review methodology are a potential lack of comprehensiveness, or an increased risk of bias. These risks are compensated for by the involvement of content experts who can ensure that the results are complete, and that they resonate with practice knowledge. RRR also incorporates local knowledge users and links to commissioning agencies, thus enhancing both usability and utilization of results.

Rapid Realist Reviews are scalable with respect to involvement of external experts based on the time and resources available. The RRR methodology works well in contexts where there is limited evidence due to its incorporation of practice evidence via expert panels.

While the end products of RRRs focus less on theory development than on intervention-outcome links, they still rely on program theories to explain how and why certain families of interventions may produce outcomes of interest within a given context for specific populations. It can be more difficult to engage policy makers in theory development, but this does not lessen the utility of realist reviews for producing evidence-informed policy. Government agencies interested in using a RRR methodology will likely need to involve expert methodologists. However, this helps maximize the efficiency of key agency or government staff.

In conclusion, for policy makers faced with making difficult decisions in short time frames, related to system-level programs/initiatives/directions, for which there is sufficient (if limited) published/research and practice-based evidence available, RRR provides a practical, outcomes focused knowledge synthesis method.

## Competing interests

The Rapid Realist Review methodology described in this paper is designed to bridge multisectoral barriers to knowledge use. Although the authors have academic interest in this methodology, they also engage in the methodology as consultants on a range of projects. Some of these projects are funded by grants managed by universities, some through contracts to the InSource Research Group. InSource was created to provide a vehicle for responding to policy maker needs for knowledge synthesis in a more timely way than is possible through the normal grant funding process.

In the interests of transparency and to address conflict of interest concerns, it is very possible that publication of this paper could enhance InSource’s reputation and could result in future contracts for the company. It must also be noted that two of the four authors (JB and AB) are InSource directors, and the other two (JS and CW) have worked, or in the future may work, under contract to InSource.

In sum, the authors may receive financial gain in the future from the publication of this manuscript. In our view, the conflict between academic and business interest in this area is unavoidable, and bridging the gap between these interests is vital to supporting the research to policy and practice process. The evidence is clear that knowledge uptake is poor without effective structures to support the process.

## Authors’ contributions

JS made substantial contributions to: the acquisition, analysis and synthesis of data presented in Table 1; designing, drafting and revising the manuscript. CW made substantial contributions to: the design, drafting and revision of the manuscript; analysis and interpretation of data in Table 1. JB made substantial contributions to: the acquisition, analysis and synthesis of data presented in Table 1; drafting and revising the manuscript. AB made substantial contributions to: the analysis and synthesis of data presented in Table 1; drafting and revising the manuscript. All authors have approved the final version of this manuscript.
